# Atrial fibrillation screening in Syrian patients reporting to the emergency department during the ongoing conflict: a cross-sectional study

**DOI:** 10.3389/fcvm.2025.1512558

**Published:** 2025-02-20

**Authors:** Ibrahim Antoun, Alkassem Alkhayer, Aref Jalal Eldin, Alamer Alkhayer, Ibrahim Salama, Khaled Yazji, Riyaz Somani, G. André Ng, Mustafa Zakkar

**Affiliations:** ^1^ Department of Cardiovascular Sciences, University of Leicester, Leicester, United Kingdom; ^2^ Faculty of Medicine, University of Aleppo, Aleppo, Syria; ^3^ Department of Medicine, University of Tishreen's Hospital, Latakia, Syria; ^4^ Department of Intensive Care, Guys and St Thomas Hospital, London, United Kingdom; ^5^ Department of Cardiology, The View Hospital, Doha, Qatar; ^6^ Department of Cardiology, University Hospitals of Leicester NHS Trust, Glenfield Hospital, Leicester, United Kingdom; ^7^ NIHR Leicester Biomedical Research Centre, Department of Research, Leicester, United Kingdom; ^8^ Department of Cardiac Surgery, University Hospitals of Leicester NHS Trust, Glenfield Hospital, Leicester, United Kingdom; ^9^ Faculty of Medicine, University of Damascus, Damascus, Syria

**Keywords:** atrial fibrillation, ECG, screening, Syria, conflict

## Abstract

**Background:**

Atrial fibrillation (AF) is the most common arrhythmia worldwide. Data regarding AF screening in conflict countries’ emergency departments (ED) is lacking.

**Methods:**

We included consecutive patients >40 years old who reported to the ED of a Syrian tertiary centre between July 2024 and September 2024. Patients had routine 12-lead electrocardiograms (ECGs) regardless of presenting complaints. Two cardiology consultants blindly verified ECG findings. We excluded critically unwell patients and ECG discrepancies between the two consultants. Data were taken from patients’ medical notes.

**Results:**

The final analysis included 694 patients, 101 (15%) had AF on the ECG. The most common presenting complaints and ECG abnormality were trauma (44%) and sinus tachycardia (15%), respectively. Compared to the rest of the patients, AF patients were older (66 vs. 59 years; *p* < 0.001), had a lower proportion of males (39% vs. 54%; *p* = 0.01), a higher prevalence of diabetes mellitus (49% vs. 21%; *p* = 0.01), and more cases of congestive cardiac failure (CCF) (38% vs. 17%; *p* < 0.001). AF patients also had a higher CHA₂DS₂-VASc score (3 vs. 2; *p* < 0.001). CCF [odds ratio [OR]: 3.3, 95% confidence interval [CI]: 1.5–6.4, *p* < 0.001] and a higher CHA₂DS₂-VASc score(OR: 4, 95% CI: 1.6–7.7, *p* < 0.001) were independently associated with positive AF screening.

**Conclusion:**

15% of patients reporting to this Syrian ED had positive AF screening. CCF and CHA₂DS₂-VASc scores are predictive of AF.

## Introduction

Atrial fibrillation (AF) is the most common type of arrhythmia worldwide, and its prevalence in middle—to low-income countries is underestimated (1). AF in the developed world is well studied. Still, there is little data on AF management and demographics in the Middle East, with only four data registries (2). AF-related research in Arab countries contributes only 0.7% of AF research worldwide (3).

Syria has been suffering from a conflict since 2011. It has been deprived of healthcare funding and resources, particularly exacerbated during the cholera and COVID-19 outbreaks (4, 5). Therefore, less than 50% of its hospitals operate as usual, with more than half its healthcare workforce forced to leave the country due to conflict (6). AF management in hospitals during the current economic and political turmoil is unclear, with a scarcity of published inpatient figures and outcomes originating from Syrian healthcare (7–10). Although late advances in AF management have enhanced the AF burden and symptom control, readmission rates continue to increase and have been one of the primary sources of AF-related financial constraints on healthcare economies around the world. One crucial problem is the fact that up to around one-third of AF may be asymptomatic (11), and this may account for a large proportion of the 20% of patients who suffer a stroke without any apparent underlying cause. In a previous study, subclinical atrial tachyarrhythmia detected by implanted devices (defibrillators or pacemakers) was associated with a more than two-fold increase in thromboembolic events (12). In the developed world, opportunistic screening for AF has been explored in pharmacy settings and primary care (13, 14). However, this has proven challenging in a conflict setting such as Syria, where the main patient pool is inside hospitals admitted through the emergency department (ED). Therefore, we aimed to explore the feasibility of opportunistic AF screening in a tertiary Syrian ED department to assess the prevalence of undiagnosed AF.

## Methods

### Study design and data collection

This single-centre cross-sectional observational study was conducted at Tishreen's University Hospital, Latakia, Syria. It is a large teaching hospital and tertiary care centre with around 860 beds. On average, the hospital provides free healthcare to approximately 50,000–60,000 inpatients yearly, with an even more significant number of outpatients seeking care in various medical departments. The ED has around 50 beds across different units, including triage, critical care, and observation. The study included patients over 40 years old reporting the ED between the 1st of July 2024 and the 1st of September 2024. A 12-lead ECG was conducted routinely regardless of the presenting complaint. Two general cardiology consultants blindly reviewed ECGs. We excluded patients with a critical condition or hemodynamic instability, and patients with discrepancies in ECG diagnosis between the two cardiology consultants. ED and medical clinical charts were examined for patients’ demographics. The research reported in this article adhered to the Declaration of Helsinki. The project was conducted as part of an audit approved by the hospital board and involved prospective analysis of anonymised collected data (reference: 277/C). The reporting of this observational study followed the Strengthening the Reporting of Observational Studies in Epidemiology (STROBE) guidelines (15).

### Study outcomes

The study's primary outcomes included the prevalence of AF in patients presenting to ED. A secondary analysis explored the correlation between demographics and the presence of AF.

### Statistical analysis

Continuous variables are expressed as median and interquartile range (IRQ). Categorical variables are expressed as counts and percentages (%). Pearson's *χ* 2 or Fisher's exact test was used for categorical variables between groups. Students’ *t*-tests and Kruskal–Wallis tests were used to compare continuous variables between the groups depending on the normality of the distribution. We used logistic regression to examine the relationship between demographics and AF presence. Our multivariable model was constructed *a priori* and included demographics that are statistically significant in the univariable model. A 2-sided *p*-value <0.05 was considered statistically significant. Statistical analysis was performed using GraphPad Prism V10.3 for Mac (San Diego, California, USA).

## Results

### Patients characteristics and primary outcomes

**Figure 1:**
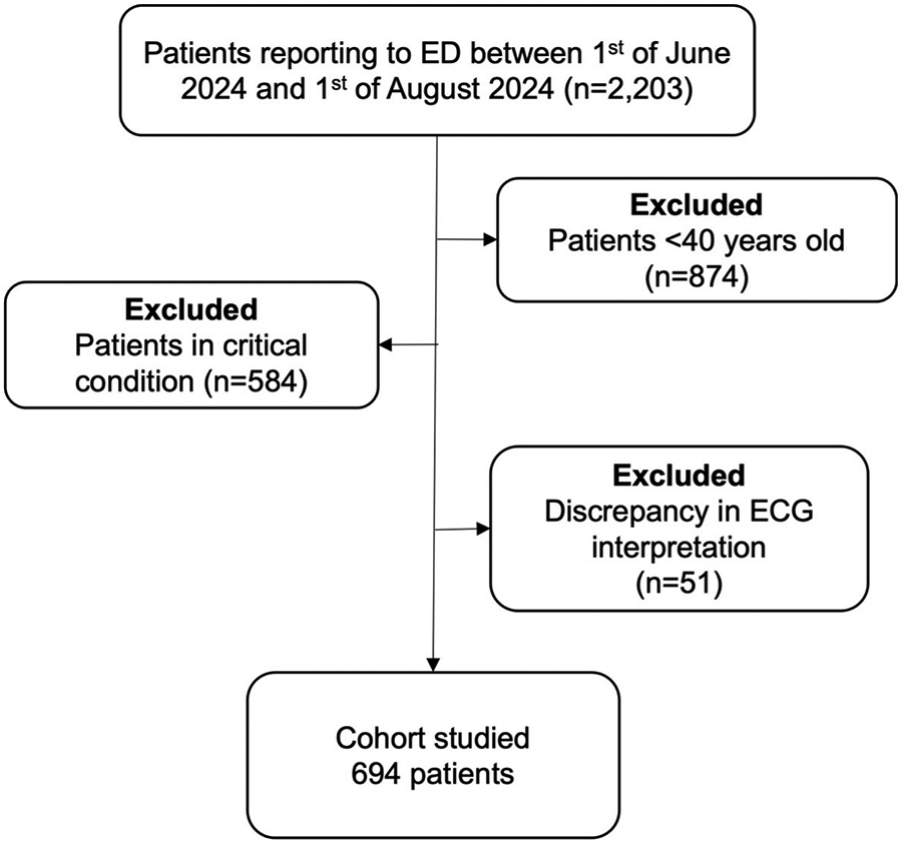
Flow chart of the patient selection criteria. AF, atrial fibrillation; ED, emergency department.

**Figure 2:**
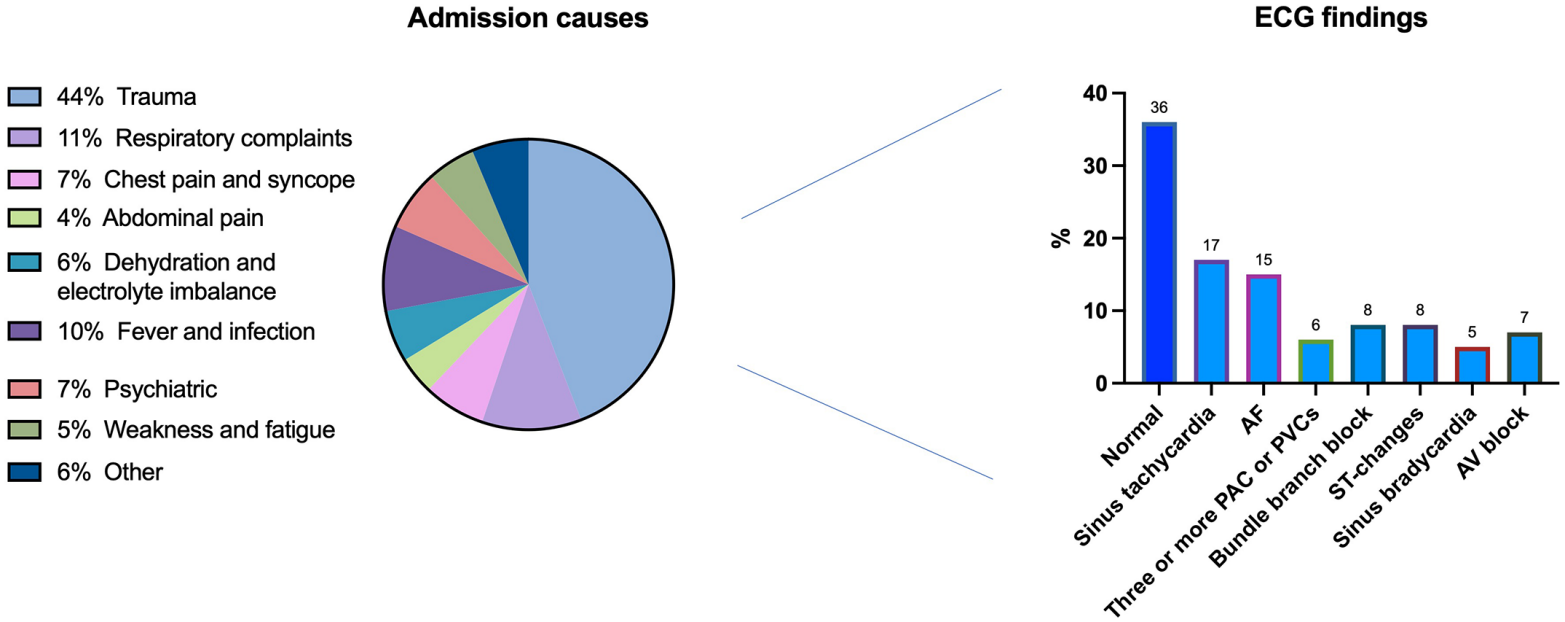
Admissions aetiology and electrocardiogram results. ECG, electrocardiogram; AF, atrial fibrillation; PAC, premature atrial contraction; PVC, premature ventricular contraction.

After applying the selection criteria in Figure 1, 694 patients were included in the final analysis, of which 101 had unknown AF proven on the 12-lead ECG (15%). Demographics are demonstrated in Table 1. Males comprised 57% of the cohort, with a median age of 61 (IQR: 52–68 years). Compared to the rest of the patients, AF patients were older (66 vs. 59 years; *p* < 0.001), had a lower proportion of males (39% vs. 54%; *p* = 0.01), a higher prevalence of diabetes mellitus (49% vs. 21%; *p* = 0.01), and more cases of congestive cardiac failure (CCF) (38% vs. 17%; *p* < 0.001). This resulted in a higher CHA₂DS₂-VASc score in AF patients [3 [2–4] vs. 2 [1–3]; *p* < 0.001]. Other ECG findings and patients’ presenting complaints are shown in Figure 2. ECG was normal in 247 (36%). The most common ECG abnormality was sinus tachycardia in 115 patients (17%), and the most common presenting complaints were trauma (44%), followed by respiratory complaints (12%) and chest pain/syncope (7%).

**Table 1:** Demographics and characteristics of patients stratified by 12-lead electrocardiogram results.

	Overall (*n* = 694)	AF (*n* = 101)	No AF (593)	*p*-value
Demographics, *n* (%) or median (IQR)				
Age (years)	61 (52–68)	69 (65*–*71*)*	59 (50–66)	<0.001
Male	361 (52%)	39 (39%)	322 (54%)	0.02
Cardiovascular comorbidities, *n* (%) or median (IQR)				
Hypertension	226 (33%)	31 (31%)	195 (33%)	0.62
Ischemic heart disease	107 (15%)	19 (19%)	88 (15%)	0.42
Diabetes mellitus	176 (25%)	49 (49%)	127 (21%)	<0.001
Cerebrovascular disease	133 (19%)	23 (22%)	110 (19%)	0.54
Congestive cardiac failure	136 (20%)	38 (38%)	98 (17%)	<0.001
Peripheral artery disease	98 (14%)	19 (19%)	79 (13%)	0.26
CHA₂DS₂-VASc score	2 (1–3)	3 (2–4)	2 (1–3)	<0.001
PCI within the last year	33 (5%)	2 (4%)	31 (5%)	0.84
CABG within the last year	18 (3%)	5 (5%)	13 (2%)	0.24
Thyroid disease	28 (4%)	6 (6%)	23 (4%)	0.78
Other comorbidities, *n* (%)				
Anaemia	106 (15%)	14 (14%)	92 (16%)	0.75
Active malignancy	51 (7%)	7 (7%)	44 (7%)	0.91
Chronic liver failure	50 (8%)	2 (4%)	48 (8%)	0.15
Chronic lung disease	73 (11%)	7 (7%)	66 (11%)	0.25
Chronic kidney failure	64 (9%)	11 (11%)	53 (9%)	0.35

CABG, coronary artery bypass graft; PCI, percutaneous coronary intervention.

### Secondary outcomes

Logistic regression models are demonstrated in Table 2. Univariable regression showed that older age [odds ratio [OR]: 2, 95% confidence interval [CI]: 1.2–3.8, *p* = 0.02], females (OR: 2.2, 95% CI: 1.2–4.1, *p* = 0.01), DM (OR: 2.3, 95% CI: 1.2–4.2, *p* = 0.004), CCF (OR: 3.1, 95% CI: 1.5–5.7, *p* = 0.01) and a higher CHA₂DS₂-VASc score (OR: 2, 95% CI: 1.6–2.5, *p* < 0.001) were associated with positive AF screening. The multivariable logistic regression model demonstrated that CCF (OR: 3.3, 95% CI: 1.5–6.4, *p* < 0.001) and a CHA₂DS₂-VASc score (OR: 4, 95% CI: 1.6–7.7, *p* < 0.001) were independently associated with positive AF screening.

**Table 2:** Logistic regression model regarding predictors of atrial fibrillation in the study.

Variables	Univariable analysis		Multivariable analysis	
	OR (95% CI)	*p*-value	OR (95% CI)	*p*-value
Age (per year increase)	2 (1.2–3.8)	0.03	1.6 (0.5–4.9)	0.09
Sex (female compared to male)	2.2 (1.2–4.1)	0.01	1.5 (0.7–3)	0.3
Diabetes mellitus (yes vs. no)	2.3 (1.2–4.2)	0.003	1.7 (0.8–29)	0.08
Congestive cardiac failure (yes vs. no)	3.1 (1.5–5.7)	<0.001	3.3 (1.5–6.4)	<0.001
Peripheral vascular disease (yes vs. no)	1.5 (0.7–3.3)	0.1		
Cerebrovascular event (yes vs. no)	1.2 (0.7–2.6)	0.6		
Ischemic heart disease (yes vs. no)	1.3 (0.6–2.8)	0.27		
Hypertension (yes vs. no)	1.3 (0.6–2)	0.55		
CHA₂DS₂-VASc score (per 1 point increase)	2 (1.6–2.5)	<0.001	4 (1.6–7.7)	<0.001
Chronic lung disease (yes vs. no)	1 (0.9–1)	0.75		
Thyroid disease (yes vs. no)	1.2 (0.5–3.9)	0.67		
Anaemia (yes vs. no)	1.1 (0.8–2.2)	0.59		
Active malignancy (yes vs. no)	1.1 (0.9–1)	0.88		
Chronic liver failure (yes vs. no)	1.3 (0.8–2)	0.62		
Chronic lung disease (yes vs. no)	1 (0.9–1)	0.95		
Chronic kidney failure (yes vs. no)	1.1 (0.8–1.2)	0.72		

OR, odds ratio; CI, confidence interval.

## Discussion

This is the first study describing the utility of opportunistic ED-based screening for AF in Syria and the Middle East. It highlights two significant novel findings for the Syrian-Arabic population: 15% had a positive screening for AF, and the CHA_2_DS_2_-VASc score and CCF were independently associated with positive AF screening.

As AF is known to have enormous implications on economies worldwide (16), recent studies have focused on many aspects of AF, including hospitalisation and readmission rates (17, 18). AF screening can identify more cases, but there is limited evidence on how it affects health outcomes. Anticoagulation treatment was associated with a reduced risk of mortality and first stroke, but it also increased the risk of significant bleeding. Therefore, the exact extent of this risk is uncertain, and no trials have assessed the benefits and risks of anticoagulation specifically for screen-detected populations (19).

Previous studies have reported conflicting results on the role of ECG screening for AF detection. For example, Fitzmaurice et al. demonstrated that both systematic and opportunistic screening in primary care increased AF detection rates compared to routine practice, with no significant difference between the two approaches (20). However, this study highlighted that systematic screening required more resource allocation without significantly improving detection rates, raising questions about cost-effectiveness. On the other hand, Morgan et al. showed that systematic nurse-led screening achieved significantly higher detection rates than opportunistic screening (4.5% vs. 1.3%) (21). This finding underscores the role of healthcare professionals in enhancing AF detection, though variability in results across practices and concerns about scalability in low-resource settings remain.

Lowres et al. further demonstrated the utility of modern technology such as iECGs, which yielded a fourfold increase in AF detection compared to routine care. However, despite improved AF diagnosis, no significant reduction in clinical events (e.g., stroke) was observed, and the high cost of $10,780 per diagnosis limits its applicability, especially in resource-limited or conflict-affected settings (22). The US Preventive Services Task Force statement 2018 concluded that evidence was insufficient to assess the benefits and harms of AF screening with routine ECGs (23). However, these data were pulled from countries without conflict and with fully functional healthcare. In a conflict country, including Syria, an ED visit is an opportunity to screen for common conditions in adults and the elderly population, including AF.

Our positive AF detection of 15% far exceeds rates reported in the developed world. For example, a multicentral US-based study showed an AF prevalence of 1.1% in the ED-based screening (24). There was no previous data from a developing world country undergoing conflict for comparison. Our results can be compared to findings from various studies conducted in developing countries. This comparison is essential to understand AF's prevalence and detection rates in different healthcare settings, particularly in low-resource environments. AF prevalence in the developing world was proposed to be between 1% and 7.4% in the general population and between 1% and 56% in hospital settings, keeping with our rate (25, 26). In rural India, a community-based screening program revealed a high burden of unrecognized AF, with detection rates significantly influenced by the methodology employed. Soni et al. reported that their innovative approach, which involved randomised home-based serial screening of individuals aged 50 years and older, yielded a higher AF detection rate than previous studies that utilised a single rhythm check (27). This suggests that the detection rate can vary significantly based on the screening strategy, which may also apply to the Syrian context. Moreover, a study conducted in Sweden found a total AF prevalence of 12.3% in a screened population, indicating that similar screening efforts in different geographical locations can yield comparable detection rates (28). In another study focusing on low-resource settings, Evans et al. emphasised the feasibility of using mobile ECG technology for AF screening, which detected a significant proportion of AF cases that would otherwise remain undiagnosed (29). This suggests that the integration of technology in screening processes can enhance detection rates, potentially leading to a higher prevalence being reported in similar settings. This comparison underscores the importance of addressing resource limitations and the compounded impact of conflict on healthcare delivery, highlighting the need for tailored strategies to improve AF screening in such settings.

Our higher AF rates may partially be attributed to the Syrian conflict, which has been ongoing since 2011 and has massively affected health infrastructure. It resulted in a high turnover of skilled staff and inadequate nurses and allied health professionals (30). Only half of the country's hospitals and primary healthcare centres are fully functional (30). Although there was no data before the conflict for comparison, the current data is likely to reflect the current state of play throughout this war-torn country. This included reduced access to preventive healthcare and a higher burden of cardiovascular risk factors and disruptions in healthcare infrastructure, which likely contributed to our high AF pickup rate.

Furthermore, socioeconomic disparities may affect the ability of patients to adhere to management plans and to attend follow-up appointments. Financial constraints may also limit access to necessary interventions and medications, leading to patients developing complications from the patient's cardiac conditions (31). Furthermore, the high AF prevalence observed in this study may also be influenced by conflict-specific factors, such as psychological stress and trauma, which are known contributors to cardiovascular disease. However, due to logistical constraints in the ED, data on these variables could not be collected. Future studies should address this gap by integrating psychosocial and conflict-related stressors assessments into the study design. CCF and CHA₂DS₂-VASc scores are known predictors of AF (32, 33), and our study's findings align with those. CCF contributes to changes in the atria, increasing the prevalence of AF due to mechanical stress, activation of certain hormones, and inflammation (33). The components of the CHA₂DS₂-VASc score, particularly CCF, indicate the shared risk factors for stroke and the development of AF. The more risk factors a patient has (as noted in the CHA₂DS₂-VASc score), the greater the likelihood of developing AF. Therefore, policymakers should put a strategy to screen these patients for AF as they appear to be at a higher risk in our Syrian cohort. Our study was limited to three months due to logistical constraints and the reliance on real-time prospective data collection to ensure accuracy. However, extending the study period to a full year could provide more comprehensive data, accounting for seasonal variations in ED presentations. Future studies should consider longer data collection durations to capture a broader spectrum of AF cases and patient demographics.

This study offers new insights into the prevalence and predictors of AF in a conflict-affected environment, emphasising the feasibility of opportunistic AF screening in EDs. It highlights the necessity for further research to examine the broader impact of conflict-specific factors, such as psychological stress, on cardiovascular health. Furthermore, the findings pave the way for larger, multi-centre studies investigating AF prevalence and management in other conflict- or low-resource settings, thereby contributing to the global understanding of AF in underrepresented populations. From a clinical standpoint, this study demonstrates that routine ECG screening in the ED can effectively identify undiagnosed AF, even in resource-limited and conflict-affected environments. Identifying CHA_2_DS_2_-VASc score and CCF as independent predictors of AF can help clinicians stratify high-risk patients for targeted screening and early intervention. This approach has the potential to reduce stroke risk and improve outcomes in underserved populations by facilitating timely anticoagulation therapy and other appropriate management strategies.

## Limitations

Data collection was limited to a single tertiary care centre in Latakia. This city was relatively less affected by the Syrian conflict than the other northern and eastern regions of Syria. Therefore, our results might not be generalisable to other centres/regions, given the significant heterogeneity in the quality and level of hospital supplies and staffing. Additionally, our analysis included only routinely collected data within the medical records and by the number of patients who presented to the hospital. Therefore, other variables potentially impacting AF prevalence may have yet to be identified. Our ECG screening might have missed paroxysmal AF and subclinical AF patients. Thus, AF prevalence might have been underestimated. This study's duration was limited to three months, which may have introduced seasonal biases in patient presentations. Additionally, expanding the study retrospectively or prospectively for a full year could yield more representative data on AF prevalence and its associated risk factors. This remains a key area for future research.

## Conclusion

Opportunistic AF screening in Syrian EDs could play a crucial role in identifying AF and initiating appropriate treatment, particularly in a conflict setting where access to routine medical care is significantly limited. A higher CHA₂DS₂-VASc score and the presence of CCF were independently associated with the presence of AF.

## Data Availability

The raw data supporting the conclusions of this article will be made available by the authors, without undue reservation.
